# Supplementary material for: Framework and algorithms for identifying honest blocks in blockchain
Source: PLoS One. 2020 Jan 8;15(1):e0227531. doi: 10.1371/journal.pone.0227531 (PMC6949114; doi:10.1371/journal.pone.0227531)
Supplement: S1 Supporting Data — (ZIP) [file pone.0227531.s001.zip › MAXCORD data/Data description.doc]

We give the data we used in the paper in the forms of RData which are put in four files for Fig 2 to Fig 5 respectively. All blockDAG networks were simulated by the R package BlockSim which can be found at <https://github.com/wulingyun/BlockSim>.

Here we give a brief introduction about these data.

maxdiscord.RData

There are a matrix and a list which are for Fig 2. The list includes all simulated blockDAG networks. The length of the list is 8 and each element represents a kind of attack power cases. In every case, there are 100 lists which represent 100 simulations. The matrix named maxdisocrd which is to show the relationship between the block creation rate and the maximum honest block discord. The rows of the matrix represent different values of the block creation rate. The columns of the matrix represent different simulation results under this kind of block creation rate case.

MAXCORD1.RData

There are two lists and twelve matrices which are for Fig 3. The two lists respectively represent the blockDAG simulation results under the moderate and strong attack power cases. The length of each list is 100 and each element represents a kind of the blockDAG simulations. The 12 matrices respectively represent the precision, recall, running time of MAXCORD-IFA and MAXCORD-EXA when the two algorithms are applied to the blockDAG simulations. The matrices related to MAXCORD-IFA are 6*100. The rows represent different values of parameter alpha. The columns represent the different simulations under this value of parameter alpha. The matrices related to MAXCORD-EXA is 1*100. The columns represent the different simulations.

MAXCORD2.RData

There are two lists and twelve matrices which are for Fig 4. The two lists respectively represent the blockDAG simulation results under the moderate and strong attack power cases. The length of each list is 100 and each element represents a kind of the blockDAG simulations. The twelve matrices respectively represent the precision and recall of MAXCORD-IFA, MAXCORD-EXACT and MAXCORD-GREEDY when the three algorithms are applied to the blockDAG simulations. These matrices are all 3*100. The rows represent different values of parameter d. The columns represent the different simulations under this value of parameter d.

MAXCORD v.s. PHANTOM.RData

There are four matrices and one list which are for Fig 5. The list includes all simulated blockDAG networks. The length of the list is 8 and each element represents a kind of attack power cases. In every case, there are 10 lists which represent 10 simulations. The four matrices respectively represent the precision and recall of MAXCORD-IFA and PHANTOM when the two algorithms are applied to these simulated blockDAG networks. The rows of the matrix represent the different kind of attack power cases. The columns of the matrix represent different simulation results under this kind of attack power case.
