# Supplementary material for: Framework and algorithms for identifying honest blocks in blockchain
Source: PLoS One. 2020 Jan 8;15(1):e0227531. doi: 10.1371/journal.pone.0227531 (PMC6949114; doi:10.1371/journal.pone.0227531)
Supplement: Attachment — Submitted filename: ResponseLetter.doc [file pone.0227531.s002.doc]

Response to Reviewer Comments

We truly appreciate the editor and reviewers for their thoughtful comments and suggestions on our manuscript entitled “Framework and algorithms for identifying honest blocks in blockchain”. We have studied reviewer’s comments carefully and presented a point-to-point response to the comments in blue.

**Reviewer #1:**

***It is a very interesting article where authors had the objective presents a novel universal framework MaxCord based on graph theory for identifying the honest blocks in BlockDAG. The authors suggest that their study is pioneering and new research needs to be done to develop a better model for MaxCord. This article is well written, results are considerable.***

Thank you very much for your positive comments. The major contribution of our manuscript is the novel universal framework MaxCord for the honest block identification problem and provides important basis for future research on the next generation of blockchain.

**Reviewer #2:**

***This paper present a new algorithm to recognize honest blocks in the blockchain technology. This problem is timely with a great importance. Using the generalization of max independent set problem seems very interesting. The English needs improvement.***

Thank you very much for your positive comments. We carefully revised the manuscript to improve the language and writing.

***The following are my comments and suggestions if the paper is selected for publication:***

1. ***The authors correctly identified the scalability and low transaction rate as key challenges of blockchain. However, it only applies to BitCoin blockchain with proof of work (PoW) mining methods and there exist a lot of other mining methods such as Proof of Stake (PoS), hybrid PoW/PoS, Proof of Importance (PoI), Delegated Proof of Stake (DPoS), PoL (Proof of Luck), and Stellar Consensus Protocol (SCP) to accelerate the block validation process that is not mentioned in the introduction.***

Thank you for your valuable suggestion. As long as the fork occurs under the implement of the consensus mechanism, the scalability and low transaction rate is the main bottleneck for the development of blockchain even if these new consensus mechanisms may accelerate the block validation process.

Under the consensus mechanism of PoW, miners strive to solve the hash function to compete for the accounting rights. The fork occurs owing to the delay of information propagation and so on. Under the consensus mechanism of PoS, the node whose product of the number of coins he owned and the time of holding these currencies is larger is easier to get the accounting rights. The block validation process is speeded up, but the fork is easier to occur because mining does not require computational power and miners can mine blocks on fork chains simultaneously. The consensus mechanism of hybrid PoW and PoS combines the advantages of the two consensus mechanisms, and it would be energy-saving and fast in confirmation. But the fork is still inevitable. Under the consensus mechanism of DPoS, there is a voting system. The coin owners would vote to choose the nodes to own the accounting rights. Though it speeds up the confirmation, the security decreases and forks occur. Under the consensus mechanism of Proof of Importance (PoI), each account (node) is assigned an importance score. Accounts with higher importance scores have higher probabilities of harvesting a block. It is similar to PoS. Under the consensus mechanism of Proof of Luck (PoL), TEE (trusted execution environments) platform’s random number generation is used to choose a consensus leader, which offers low-latency transaction validation, deterministic confirmation time and negligible energy consumption, but forks still happens. The Stellar Consensus Protocol (SCP) provides a way to reach consensus without relying on a closed system to accurately record financial transactions. SCP is the first provably safe consensus mechanism that simultaneously enjoys four key properties: decentralized control, low latency, flexible trust, and asymptotic security, but forks still occur.

In light of your valuable suggestion, we added the following sentence in the manuscript to mention these new consensus mechanisms and their influence on the scalability:

“Many other consensus mechanisms such as Proof of Stake (PoS), Delegated Proof of Stake (DPoS), Proof of Importance (PoI), Proof of Luck (PoL), hybrid PoW/PoS, were proposed to accelerate the block validation process, but the performance of blockchain is still not substantially improved due to the occurrence of forks. Moreover, the consensus mechanisms with higher throughput often sacrifice the decentralization to some extent.”

2. ***It seems to me that the BlockDAG is somewhat related to the Tangle structure that is considered to (or at least is claimed to) replace the blockchain structure in the next generation of blockchains to solve the scalability issue. For example, see the following reference. Please clarify on this matter.***

Thank you for your valuable comment. Tangle is also based on the directed acyclic graph (DAG) structure and therefore it can also alleviate the scalability issue of blockchain. But there are some differences between Tangle and BlockDAG.

First, the vertexes of the Tangle DAG structure are individual transactions instead of blocks (therefore sometimes called Blockless). Each new transaction is linked to only two preceding tip transactions (i.e. the transactions not referenced by any other transactions) which are chosen based on MCMC (Markov Chain Monte Carlo) random walk and cumulative weights. The weight of a transaction is proportional to the amount of work that the issuing node invested into it. Tangle attempts to distinguish the “dishonest” transactions from normal transactions by the tip selection algorithm, and the “dishonest” transaction may fall into oblivion. This mechanism is somehow similar to the classical blockchain in which the main chain is selected by the longest-chain rule and the blocks outside the main chain will be ignored.

While in the BlockDAG, the new block could be connected to all the tip blocks observed by the node when issuing the new block. All blocks are kept in the system and can be referenced directly or indirectly by the new blocks in future. BlockDAG attempts to identify the “dishonest” blocks by an algorithm independent of the construction of DAG.

In light of your comment, we added more discussion on the different approaches for DAG-based blockchain technology.

3. ***Please clarify on the “honesty” concept in the early pages of the paper. Is it equivalent to tampered blocks with false information, or blocks that are mined by illegitimate miner? The reader should wait until section 2 to get what you mean.***

Thank you for your valuable suggestion. We clarified the “honest blocks” and “dishonest blocks” in the first section as follows.

“First, distinguish the honest blocks (blocks generated and propagated timely by the miners who conform with the rules of blockchain) from the suspect dishonest blocks (blocks generated by the miners who deliberately keep the blocks secrete for a long time or take other actions which are not in accordance with the rules of blockchain).”

4. ***Please explain Equation (1). Why discord is finite when $d_{AB}^{D}$ is infinite and there is no common descendant for blocks A and B. I may miss a point here but it does not mean that B is not in the anticone of A?***

The discord of two blocks A and B is defined as the length of loop formed by the blocks A, B, their nearest common ancestor and nearest common descendant. For the situation where there is no common descendant of two blocks, a virtual block is added which references all tip blocks. Please note that $d_{AB}^{D}$ is calculated in the original DAG without the virtual block. When $d_{AB}^{D}$ is infinite, B is indeed in the anticone of A (since B is neither in the future nor in the past of A) and A is also in the anticone of B. $d_{AB}^{D}$ is infinite also implies there is no common decedent for blocks A and B in the original DAG. Therefore, the discord of blocks A and B is calculated using the virtual block, which is their only common decedent in the modified DAG with virtual block, as the sum of $d_{AB}^{A}$, $d_{A}^{T}$, $d_{B}^{T}$ and 2, where $d_{A}^{T}$ and $d_{B}^{T}$ represents the shortest distance between A, B and their nearest tip block respectively, and 2 is the total distance between the tip blocks and the virtual block. If B is not in the anticone of A, A and B are connected and their discord should be zero (the first case in Equation (1)).

In light of your comments, we revised the manuscript to make the definition of discord much clearer.

5. ***Please explain why the discord is smaller than 10 for two honest blocks. First, why it is larger for dishonest blocks and secondly where the threshold 10 coming from. Does it depend on the size of networks, transaction rates, etc?***

The discord of two blocks A and B attempts to estimate the temporal inconsistency (ambiguity) between two blocks. If the block A is in the past of B or vice versa, their temporal order is unambiguous. Thus, the discord of A and B is defined as zero. If two blocks are in the anticone of each other, their temporal order cannot be determined. But the time discrepancy of two blocks are bounded by their nearest common ancestor and nearest common descendant, since the real creation time of a block is bounded by its ancestors and descendants. In fact, the discord of two blocks is a simple but reliable measure of the time discrepancy between their nearest common ancestor and nearest common descendant.

The dishonest blocks always intend to hide or counterfeit its real creation time in order to carry out some attack such as double spending. Therefore, the discords between the dishonest block and most honest blocks should be very large, otherwise its real creation time would be bounded by some honest blocks into a small interval. For example, the attackers either keep their blocks secret for a long time or only refer to their own blocks, which leads very large time discrepancy between the nearest common ancestor and the nearest common descendant of the dishonest block and most honest blocks.

On the other hand, the discord of two honest blocks is normally much smaller. If the links between blocks are not artificially manipulated, the discord of two blocks should only depend on the network propagation speed (which further depends on the network bandwidth and the block size) and the block creation rate. When the network propagation speed or the block creation rate increases, the time discrepancy between the nearest common ancestor and the nearest common descendant will decline, however, the length of shortest path between two blocks will increase and cancel out the decline of time discrepancy to some extent. Therefore, the discords are not very sensitive to the network propagation speed and the block creation rate, which is confirmed by our simulation results.

The threshold 10 comes from the simulation results as shown in Fig 2 of the manuscript. Since the precision is much more important than the recall in the honest block identification problem, it is acceptable to use a threshold slightly smaller than the real maximum discord of honest blocks.

In light of your comments, we revised the manuscript to clarify the motivation of discord.

6. ***My another question is that this screening mechanism applies to blocks after they join the graph and anticones are formed. Due to the random block selection methods, it may take a long time for a pair of two blocks to appear in each other’s anticone. Please clarify.***

It may take a long time to form the whole anticone of a block. In some extreme situation such as under attack, the anticone of a block might infinitely grow when some new blocks added to the DAG deliberately do not reference the block directly or indirectly. However, the relationship between two blocks will be determined immediately once both two blocks join the DAG. After a block is added to the DAG, the past of the block is known and fixed. If neither the block A is in the past of the block B, nor the block B is in the past of the block A, they are in the anticone of each other.

7. ***The difference between the Maxcord-IFA and Maxcord-Greedy is not clear to me.***

The Maxcord-Greedy algorithm directly solve the model MaxCord-(0,*d*), using the greedy rule to repeatedly remove the vertex (block) with the maximum degree as long as the remaining vertex set is not an independent set.

The Maxcord-IFA algorithm solves the model MaxCord-(0,*d*) by iteratively filtering out the suspect dishonest blocks identified by a series of models MaxCord-(0,*s*) with *s* > *d*. Starting with a very large *d*, it solves the maximum independent set model and filters out all suspect dishonest blocks by using the exact algorithm of the maximum independent set problem. Then it decreases the value of *s* and solves a new model MaxCord-(0,*s*) defined on the remain blocks. The procedure is repeated until *s* converges to the predetermined *d*.

In light of your comments, we revised the manuscript to clarify two algorithms.

8. ***It would be useful to introduce the simulation Environment***

Thank you for your valuable suggestion. We added some brief introduction of the simulation environment at the beginning of section 3.

9. ***The figures are barely readable. Perhaps adding some Tables would be helpful.***

Thank you for your valuable suggestion. We redrew all figures and added Table 1 to show the statistical analysis of maximum honest block discords.

10. ***The authors compared Maxcord family algorithms, but I encourage the authors to compare their method against other methods as well.***

Thank you for your advice. We have already compared Maxcord-IFA (the best in Maxcord family algorithms) with PHANTOM developed by Zohar et al. in section 3.3 (Figure 5). The comparison shows that Maxcord-IFA is an effective and efficient algorithm for the honest block identification problem.
